# Polyacetylenes from *Notopterygium incisum*–New Selective Partial Agonists of Peroxisome Proliferator-Activated Receptor-Gamma

**DOI:** 10.1371/journal.pone.0061755

**Published:** 2013-04-22

**Authors:** Atanas G. Atanasov, Martina Blunder, Nanang Fakhrudin, Xin Liu, Stefan M. Noha, Clemens Malainer, Matthias P. Kramer, Amina Cocic, Olaf Kunert, Andreas Schinkovitz, Elke H. Heiss, Daniela Schuster, Verena M. Dirsch, Rudolf Bauer

**Affiliations:** 1 Department of Pharmacognosy, University of Vienna, Vienna, Austria; 2 Institute of Pharmaceutical Sciences, Department of Pharmacognosy, Karl-Franzens-University Graz, Graz, Austria; 3 Institute of Pharmacy/Pharmaceutical Chemistry and Center for Molecular Biosciences Innsbruck (CMBI), University of Innsbruck, Innsbruck, Austria; 4 Department of Pharmaceutical Biology, Faculty of Pharmacy, Gadjah Mada University, Yogyakarta, Indonesia; 5 Institute of Pharmaceutical Sciences, Department of Pharmaceutical Chemistry, Karl-Franzens-University Graz, Graz, Austria; Broad Institute of Harvard and MIT, United States of America

## Abstract

Peroxisome proliferator-activated receptor gamma (PPARγ) is a key regulator of glucose and lipid metabolism and therefore an important pharmacological target to combat metabolic diseases. Since the currently used full PPARγ agonists display serious side effects, identification of novel ligands, particularly partial agonists, is highly relevant. Searching for new active compounds, we investigated extracts of the underground parts of *Notopterygium incisum*, a medicinal plant used in traditional Chinese medicine, and observed significant PPARγ activation using a PPARγ-driven luciferase reporter model. Activity-guided fractionation of the dichloromethane extract led to the isolation of six polyacetylenes, which displayed properties of selective partial PPARγ agonists in the luciferase reporter model. Since PPARγ activation by this class of compounds has so far not been reported, we have chosen the prototypical polyacetylene falcarindiol for further investigation. The effect of falcarindiol (10 µM) in the luciferase reporter model was blocked upon co-treatment with the PPARγ antagonist T0070907 (1 µM). Falcarindiol bound to the purified human PPARγ receptor with a *K_i_* of 3.07 µM. *In silico* docking studies suggested a binding mode within the ligand binding site, where hydrogen bonds to Cys285 and Glu295 are predicted to be formed in addition to extensive hydrophobic interactions. Furthermore, falcarindiol further induced 3T3-L1 preadipocyte differentiation and enhanced the insulin-induced glucose uptake in differentiated 3T3-L1 adipocytes confirming effectiveness in cell models with endogenous PPARγ expression. In conclusion, we identified falcarindiol-type polyacetylenes as a novel class of natural partial PPARγ agonists, having potential to be further explored as pharmaceutical leads or dietary supplements.

## Introduction

Peroxisome proliferator activated receptors (PPARs) are ligand-dependent nuclear receptors which play a major role in lipid and glucose metabolism [1]–[5]. Three subtypes of PPAR, namely PPARα, PPARβ/δ, and PPARγ, have been identified in human and other species [6], [7]. Upon ligand binding, PPARs form heterodimers with another nuclear receptor, the retinoid X receptor (RXR) and subsequently bind to response elements located in the promoter region of their target genes [8]. The binding of a PPAR/RXR heterodimer to its PPAR response element (PPRE) triggers the recruitment of nuclear receptor coactivators [9], [10], and subsequent chromatin rearrangement allowing initiation of the transcription of target genes [11].

The three PPAR subtypes are expressed in different tissues and regulate distinct physiological functions. PPARα is mainly expressed in muscle, liver, heart, and kidney, and is involved in the regulation of genes that play a role in lipid and lipoprotein metabolism [12], [13]. The PPARβ/δ subtype is ubiquitously expressed in various tissues and predominantly associated with lipid metabolism and energy expenditure [14]. Among the three PPAR subtypes, PPARγ is the best studied one. PPARγ is expressed in adipose tissue, lung, large intestine, kidney, liver, heart, and macrophages [15], and is engaged in the regulation of adipogenesis, insulin sensitivity, and inflammation [16]. The important functions of PPARγ in the regulation of glucose and lipid metabolism make it an attractive pharmacological target for combating metabolic diseases [17]–[19]. It is established that the activation of this receptor mediates the action of the glitazone-type drugs clinically used for the treatment of type 2 diabetes [20]. Furthermore, recent findings supporting a role of PPARγ in inflammation and cell growth have promoted the investigation of PPARγ agonists as experimental drugs for some chronic diseases such as atherosclerosis and cancer, among others [21]–[23]. The glitazones currently used in clinics (e.g., pioglitazone) potently activate PPARγ as full agonists. In spite of being effective drugs, their use is limited due to undesirable side effects [24], urging the identification and characterization of new PPARγ agonists. A number of recent studies revealed that partial PPARγ agonists inducing submaximal receptor activation are demonstrating great promise by exerting good hypoglycaemic activity with reduced side effects [25]–[27].

Natural products encompass a broad structural diversity of secondary metabolites, which often represent privileged structures serving a variety of biological functions [28]. Therefore, we focused on the identification of novel PPARγ activators derived from natural sources. As part of an ethnopharmacology-based screening approach, extracts from medicinal plants used in traditional Chinese medicine to treat inflammation-related conditions were tested for PPARγ activation. Extracts of the rhizomes and roots of *Notopterygium incisum* displayed significant PPARγ activation. The plant has been traditionally used in China for the treatment of rheumatism, cold, and headache [29], and its lipophilic extracts have shown inhibitory effects on 5-lipoxygenase and cyclooxygenase [30]. Pure compounds isolated from the dichloromethane extracts were now examined for PPARγ activation, leading to the identification of falcarindiol-type polyacetylenes as a novel group of natural selective partial PPARγ agonists.

## Materials and Methods

### Chemicals, Cell Culture Reagents, and Plasmids

Calf serum, L-glutamine, and Dulbecco’s modified Eagle’s medium (DMEM) containing 4.5 g/l glucose was purchased from Lonza (Basel, Switzerland). Fetal bovine serum (FBS) was supplied from Gibco (Lofer, Austria). PPARα and PPARβ/δ agonists GW7647 and GW0742, respectively, as well as the PPARγ antagonist T0070907, were purchased from Cayman (Missouri, USA); pioglitazone was purchased from Molekula Ltd (Shaftesbury, UK). All other chemicals were obtained from Sigma–Aldrich (Vienna, Austria). The tested compounds, or dried plant extracts, were dissolved in dimethyl sulfoxide (DMSO), aliquoted, and stored at −20°C until use. The final concentration of DMSO in all experiments was 0.1% or lower. An equal amount of DMSO was always tested in each experiment to assure that the solvent vehicle does not influence the results. The three human PPAR subtype expression plasmids (pSG5-PL-hPPAR-alpha, pSG5-hPPAR-beta, pSG5-PL-hPPAR-gamma1) were a kind gift from Prof. Beatrice Desvergne and Prof. Walter Wahli (Center for Integrative Genomics, University of Lausanne, Switzerland), and the luciferase reporter plasmid (tk-PPREx3-luc) was kindly provided by Prof. Ronald M. Evans (Salk Institute for Biological Studies, San Diego, California).

### PPAR Luciferase Reporter Gene Transactivation

HEK-293 cells (ATCC, USA) were cultured in DMEM with phenol red, supplemented with 100 U/ml benzylpenicillin, 100 µg/ml streptomycin, 2 mM L-glutamine, and 10% FBS. Cells were maintained in 75 cm^2^ flasks containing 10 ml medium at 37°C and 5% CO_2_. The cells were seeded in 10 cm dishes at a density of 6 × 10^6^ cells/dish, incubated for 18 h, and transfected by the calcium phosphate precipitation method [31] with 4 µg of the reporter plasmid (tk-PPREx3-luc), 4 µg from the respective PPAR subtype expression plasmid, and 2 µg green fluorescent protein plasmid (pEGFP-N1, Clontech, Mountain View, CA) as internal control. After 6 h, the transfected cells were harvested and re-seeded (5 × 10^4^ cells/well) in 96-well plates containing DMEM without phenol red, supplemented with 100 U/ml benzylpenicillin, 100 µg/ml streptomycin, 2 mM L-glutamine, and 5% charcoal-stripped FBS. The cells were further treated with the indicated extracts or compounds or the solvent vehicle and incubated for 18 h. The medium was then discarded and the cells were lysed with a reporter lysis buffer (E3971, Promega, Madison, USA). Luciferase activity of the cell lysates was evaluated using a GeniosPro plate reader (Tecan, Grödig, Austria). The luminescence signals obtained from the luciferase activity measurements were normalized to the EGFP-derived fluorescence, to account for differences in the transfection efficiency or cell number. Luciferase reporter gene assays were performed with polyacetylene concentrations up to 30 µM as higher dosages led to a downregulation of the internal normalization control (EGFP, not shown).

### Extraction and Isolation

**Figure 1:**
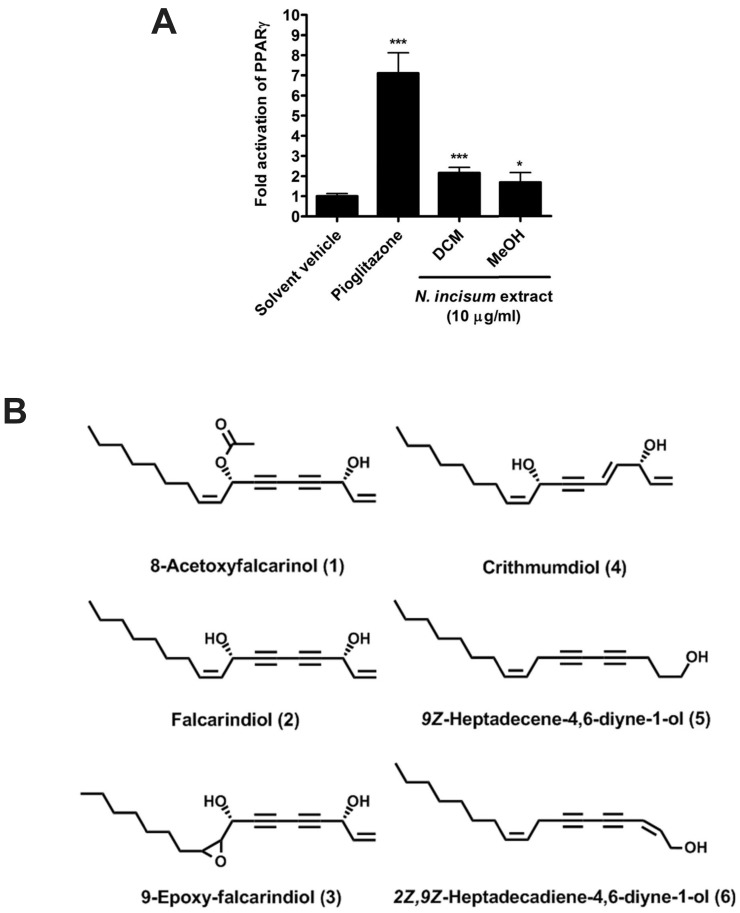
PPARγ activation by *N.* *incisum* extracts and chemical structures of the isolated polyacetylenes. (**A**) HEK-293 cells were transiently co-transfected with a plasmid encoding full-length human PPARγ, a reporter plasmid containing PPRE coupled to a luciferase reporter and an EGFP plasmid as internal control. After re-seeding, cells were treated as indicated for 18 h. Since the positive control pioglitazone (5 µM), as well as the dried DCM- and MeOH-extract were reconstituted in DMSO, cells were treated with equal amount of the solvent vehicle DMSO (0.1%) as negative control. The luciferase activity was normalized to the EGFP-derived fluorescence, and the result is expressed as fold induction compared to the solvent vehicle control. The data shown are means ± SD of three independent experiments each performed in quadruplet. ***p<0.001, *p<0.05 (compared to the solvent vehicle group; ANOVA/Bonferroni). (**B**) Chemical structures of the PPARγ-activating polyacetylenes isolated from *N. incisum*.

Plant material of *Radix et Rhizoma Notopterygii* was purchased from Plantasia Austria in 2008. A voucher specimen (No. 650107) is kept at the Institute of Pharmaceutical Sciences, Department of Pharmacognosy at the University of Graz. The plant material was grounded and extensively percolated with dichloromethane (DCM) to obtain a crude DCM extract. Bioassay-guided fractionation of the DCM extract resulted in the isolation and identification of the following compounds (Fig. 1B): 8-acetoxyfalcarinol (**1**, 3R,8S,9Z-3-hydroxyheptadeca-1,9-diene-4,6-diyne-8-yl acetate), falcarindiol (**2**, 3R,8S,9Z-heptadeca-1,9-diene-4,6-diyne-3,8-diol), 9-epoxy-falcarindiol (**3**, 1R,6R-1-(3-heptyloxiran-2-yl)octa-7-ene-2,4-diyne-1,6-diol), crithmumdiol (**4**, 3R,4E,8S,9Z-heptadeca-1,4,9-triene-6-yne-3,8-diol), 9Z-heptadecene-4,6-diyne-1-ol (**5**) and 2Z,9Z-heptadecadiene-4,6-diyne-1-ol (**6**). The purity of the isolated compounds was over 95% for **1**-**4** and above 90% for **5** and **6**. All analytical HPLC experiments were performed using an Agilent 1100 series system (Agilent 1100, Series Degaser G1311 A, Quat Pump G1311, Autosampler G1313 A, Colcom G1316 A, DAD 1315 B) equipped with a diode-array detector. Analyses were performed using a SB-C18 Zorbax column (3.5 µm; 150×2.1 mm; Agilent Technologies) at a flow rate of 300 µl/min and a gradient elution program. The extract was phytochemically investigated using a combination of different chromatographic techniques such as LC, SPE (RP-C18), and preparative HPLC (RP-C18). Isolated constituents were characterized and identified by multidimensional NMR spectroscopy (^1^H, ^13^C, COSY, HMBC, HSQC) and mass spectrometry.

### Receptor Binding Study

The LanthaScreen® time-resolved fluorescence resonance energy transfer (TR-FRET) PPARγ competitive binding assay (Invitrogen, Lofer, Austria) was performed according to the manufacturer’s protocol. The test compounds dissolved in DMSO or the blank solvent vehicle (DMSO) were incubated together with the human PPARγ ligand-binding domain (LBD) tagged with GST, a terbium-labeled anti-GST antibody, and a fluorescently labeled PPAR ligand (Fluormone Pan-PPAR Green, Invitrogen). In this assay, the fluorescently labeled ligand is binding to the human PPARγ LBD, which brings it in close spatial proximity to the terbium-labeled anti-GST antibody. Excitation of the terbium at 340 nm results in energy transfer (FRET) and partial excitation of the fluorescently labeled PPAR ligand, followed by emission at 520 nm. Test-compounds binding to the human PPARγ LBD are competing with the fluorescently labeled ligand and displacing it, resulting in a decrease of the FRET signal. The signal obtained at 520 nm is normalized to the signal obtained from the terbium emission at 495 nm; therefore, the decrease in the 520 nm/495 nm ratio is used as a measure for the ability of the tested compounds to bind to the human PPARγ LBD. All measurements were performed with a GeniosPro plate reader (Tecan, Grödig, Austria).

### Molecular Docking

To predict binding modes of the falcarindiol-type polyacetylene PPARγ partial agonists, a docking study was performed, using the quantum mechanics-polarized ligand docking (QPLD) workflow [32] in Maestro version 9.2.112 (Schrödinger, LLC, New York, NY, 2011; http://www.schrodinger.com). In this docking study, the X-ray crystal structure of PPARγ with two co-crystallized molecules of the natural product magnolol was used (Protein Data Bank entry: 3r5n; http://www.rcsb.org/pdb/) [33]. The ligand and protein preparation were conducted using the Ligprep module with default settings for the ligands and the Protein Preparation Wizard for the protein. The protein was prepared by adding hydrogens, removing water molecules, assigning atom and bond types, and a refinement by completing missing side chains. Then, an exhaustive exploration of the hydrogen bond assignment was conducted. The protein was minimized applying the OPLS-AA 2005 force field with a threshold of 0.3 Å RMSD. Afterward, atomic partial charges of the ligands were calculated within the QPLD workflow, applying the semi-empirical method AM1. Furthermore, the initial docking was conducted using Glide in standard precision (SP) mode, basically default settings, and returning up to five diverse poses for each ligand. These intermediate poses were submitted to the second docking procedure, after calculating the atomic partial charges of the ligands based upon the QM/MM approach performed by the module QSite. Mulliken population analysis was performed for the calculation of ligand atomic partial charges, which is based on the proposed orientation of the ligands relative to the receptor, and, therefore, influenced by the electrostatic field of the receptor. Afterwards, the final docking poses were retrieved using Glide in SP mode. For the final ranking of the docking poses, several scoring functions were evaluated, from which the scoring from Emodel calculations achieved the best results in several runs. To theoretically validate the docking workflow, the docking poses of magnolol were compared to its native pose in the X-ray crystal structure by calculating the geometric RMSD. The highest-ranked pose had an RMSD of 0.555 Å and the mean RMSD of all 10 calculated poses was 0.554 Å, respectively. Finally, the docking poses of falcarindiol were visually inspected and analyzed within the program LigandScout 3.03 [34] (Inte:Ligand GmbH, Maria Enzersdorf, Austria, 2012; http://www.inteligand.com).

### Adipogenicity of Falcarindiol

3T3-L1 preadipocytes (ATCC, USA) were propagated in DMEM supplemented with 10% calf serum. For differentiation, preadipocytes were grown to confluence (day −2) and kept for two additional days before the medium was changed to DMEM supplemented with 10% FBS, 1 µg/ml insulin, and falcarindiol (10 µM) or the full PPARγ agonist pioglitazone (10 µM) (day 0). The medium was renewed every two days until day 8. For an estimate of lipid accumulation, Oil Red O staining was performed. For this, cells were fixed in 10% formaldehyde for 1 h and stained with Oil Red O for 10 min. After washing off excessive dye, bound dye was solubilized with 100% isopropanol and photometrically quantified at 550 nm. For better appreciation of the adipogenic potential of the investigated PPARγ agonists, a subset of preadipocytes was treated according to a common standard differentiation protocol (2 days in DMEM/10% FBS/500 µM IBMX/500 nM dexamethasone and 1 µg/mL insulin (MDI), followed by 2 days in DMEM/10% FBS/1 µg/ml insulin and 6 days in DMEM/10% FBS).

### 2-Deoxy-D-(1H^3^)-glucose Uptake

Confluent 3T3-L1 preadipocytes (ATCC, USA) were differentiated (day 0) into mature adipocytes for 10 days (2 days in DMEM supplemented with 10% FBS, 50 µM IBMX, 1 µg/ml insulin and 500 nM dexamethasone followed by 2 days in DMEM/10% FBS/1 µg/ml insulin and 6 days in DMEM/10% FBS), and incubated with the test compounds (10 µM) or 0.1% DMSO as vehicle control for 48 h at 37°C. Before the experiment, cells were incubated for 4 h in DMEM/0.1% BSA followed by 1 h in KRH/BSA buffer (50 mM HEPES, 136 mM NaCl, 23.5 mM KCl, 1.25 mM MgSO_4_, 1.25 mM CaCl_2_ and 0.1% BSA). The cells were stimulated with insulin (500 pM) for 15 min, or not stimulated (for the “basal uptake” evaluation), and then glucose uptake was initiated by addition of 2-deoxy-D-glucose spiked with 2-deoxy-D-(1H^3^)-glucose (final concentrations 0.1 mM and 0.45 µCi/ml, respectively). After 10 min the reaction was stopped by three rapid washes with ice-cold PBS. The glucose uptake rate was determined by liquid scintillation counting (Perkin Elmer, Waltham, MA, USA) of cell lysates (20 mM HEPES, 1% Igepal CA-630), and normalized to protein content assessed by the Bradford protein assay [35].

### Novelty Evaluation

The novelty evaluation of the findings was performed with PubMed (http://www.ncbi.nlm.nih.gov/pubmed/) and SciFinder (https://scifinder.cas.org/) using “falcarindiol” as keyword, resulting in 99 and 364 literature references, respectively (as of 13^th^ of December, 2012). None of the retrieved references was related to PPAR modulation. To further evaluate the novelty of the chemical scaffold of falcarindiol as PPAR ligand, chemical similarity search was performed with SciFinder, using the query structure of falcarindiol with a Tanimoto similarity score >80%. As outcome 714 similar chemical structures, associated with 1367 literature references were retrieved. None of the obtained literature references was related to PPAR.

### Statistical Methods and Data Analysis

All statistical analyses were done with the GraphPad Prism software version 4.03. One-way analysis of variance (ANOVA) with Bonferroni post hoc test, or two-tailed paired t-test was used to determine statistical significance. Nonlinear regression (sigmoidal dose response) was used to calculate the EC_50_ values and maximal fold activation. *K_i_* values of the investigated compounds in the receptor binding assay were calculated with the Cheng-Prusoff equation: (*K_i_*) = IC_50_/(1+ L/K_D_)), where IC_50_ is the concentration of competitor that displaces 50% of the ligand, L is the concentration of Fluormone™ Pan-PPAR Green used in the assay (5 nM), and K_D_ is the binding constant (2.8 nM) of Fluormone™ Pan-PPAR Green to PPARγ LBD [36].

## Results

### Identification of Polyacetylenes from Notopterygium Incisum as Selective Partial PPARγ Agonists

Aiming to identify novel PPARγ ligands from natural sources with an ethnopharmacological background, we used a PPARγ-driven luciferase reporter gene assay (Fig. 1A) and observed a significant PPARγ activation exerted by the DCM (2.16±0.28-fold activation, p<0.001) and MeOH (1.68±0.49-fold activation, p<0.05) extracts from *N. incisum* roots and rhizomes applied at 10 µg/ml (Fig. 1A). A bioactivity-guided approach using the DCM extract led to the isolation of six active polyacetylenes: 8-acetoxyfalcarinol (**1**), falcarindiol (**2**), 9-epoxy-falcarindiol (**3**), crithmumdiol (**4**), 9Z-heptadecene-4,6-diyne-1-ol (**5**), and 2Z,9Z-heptadecadiene-4,6-diyne-1-ol (**6**) (Fig. 1B). All polyacetylenes activated PPARγ as partial agonists with maximal activation ranging from 1.73- to 3.26-fold compared to the blank treatment control, while the full agonist pioglitazone activating PPARγ up to 9.28-fold. The observed activating effect was specific for PPARγ with no activation of PPARα or PPARβ/δ (Table 1). Falcarindiol (**2**) is one of the most abundant polyacetylenes that we identified in *N. incisum*. Furthermore, it is one of the best investigated polyacetylenes in general, and is broadly found in many other plant species. Since all active compounds that we identified from *N. incisum* were polyacetylenes, and since PPARγ activating properties of this class of compounds have not been reported so far (see also “Novelty evaluation” in the “Materials and Methods” section), we have chosen the prototypical polyacetylene falcarindiol for further pharmacological characterization.

**Table 1:** Activity of the isolated polyacetylenes towards the three subtypes of human PPAR (α, β/δ, γ) in a luciferase reporter transactivation assay.

	PPARγ activation		PPARα activation		PPARβ/δ activation	
	EC_50_ (µM)	maximal foldactivation	EC_50_ (µM)	maximal foldactivation	EC_50_ (µM)	maximal foldactivation
Pioglitazone	0.41	9.28	–	–	–	–
GW7647	–	–	0.0021	3.08	–	–
GW0742	–	–	–	–	0.0017	20.2
(1) 8-Acetoxyfalcarinol	3.59	2.36	n.d.	n.d.	n.d.	n.d.
(2) Falcarindiol	3.29	3.26	n.d.	n.d.	n.d.	n.d.
(3) 9-Epoxy-falcarindiol	2.03	1.88	n.d.	n.d.	n.d.	n.d.
(4) Crithmumdiol	4.58	2.29	n.d.	n.d.	n.d.	n.d.
(5) 9-Heptadecene-4,6-diyne-1-ol	11.31	1.92	n.d.	n.d.	n.d.	n.d.
(6) *2*Z,*9*Z-Heptadecadiene-4,6-diyne-1-ol	4.18	1.73	n.d.	n.d.	n.d.	n.d.

n.d. not detected up to 30 µM.

HEK-293 cells transiently co-transfected with the respective PPAR subtype expression plasmids, a luciferase reporter plasmid (tk-PPREx3-luc), and EGFP as internal control were treated with six different concentrations of the polyacetylenes (0.1–30 µM) for 18 h. The luciferase activity was expressed as fold induction above the vehicle control (DMSO, 0.1%) after normalization to the EGFP-derived fluorescence. To verify the specificity of the performed assays, GW7647, GW0742, and pioglitazone were used as a selective agonist for PPARα, PPARβ/δ, and PPARγ, respectively. EC50 and maximal fold activation were determined with a non-linear regression in the GraphPad Prism software version 4.03 (GraphPad Software Inc, USA). The data shown are means of three independent experiments performed in triplicate.

### Transactivation Activity of Falcarindiol in the Luciferase Reporter Model

**Figure 2:**
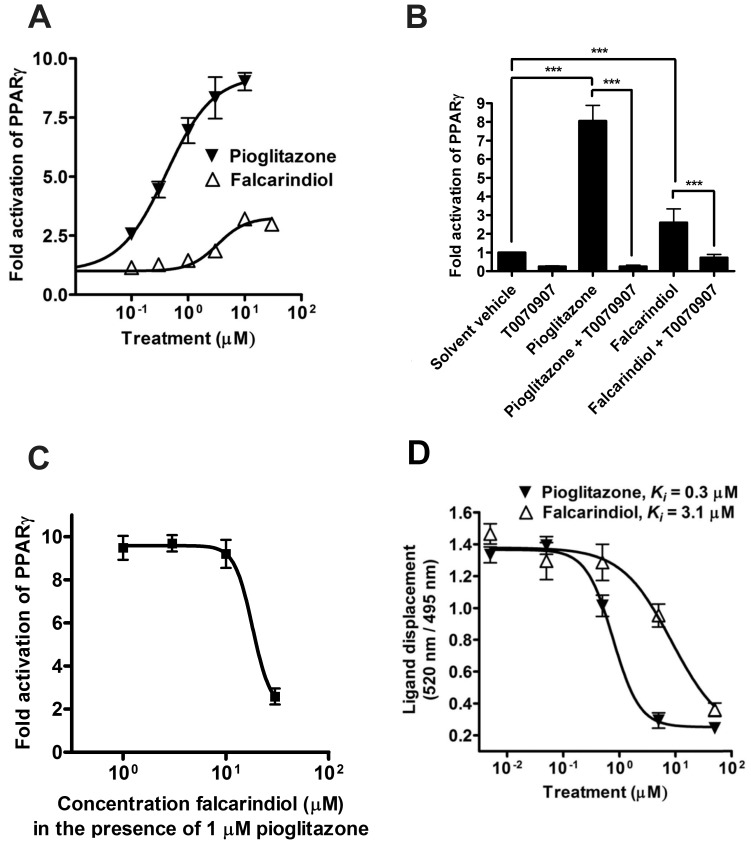
PPARγ-mediated transactivation activity as well as receptor binding activity of falcarindiol. (**A**) HEK-293 cells, transiently transfected with a human PPARγ expression plasmid, a luciferase reporter plasmid (tk-PPREx3-luc) and EGFP as internal control, were treated with different concentrations of pioglitazone or falcarindiol (0.1–30 µM) for 18 h. Luciferase activity was normalized by the EGFP-derived fluorescence, and the result is expressed as fold induction compared to the solvent vehicle control (DMSO, 0.1%). The data points shown are means ± SEM of three independent experiments each performed in quadruplet. (**B**) Cells were transfected and treated as indicated above. Pioglitazone was applied at 5 µM, falcarindiol at 10 µM, and the PPARγ antagonist T0070907 at 1 µM. The data shown are means ± SD of six independent experiments each performed in quadruplet. ***p<0.001 (ANOVA/Bonferroni). (**C**) The cells were prepared as indicated above and treated for 18 h with different concentrations of falcarindiol, always in the presence of 1 µM pioglitazone. The data shown are means ± SD of three independent experiments each performed in quadruplet. (**D**) Dilutions of the two investigated compounds were prepared in DMSO and mixed with a buffer containing the hPPARγ LBD tagged with GST, terbium-labelled anti-GST antibody, and fluorescently-labelled PPARγ agonist. After 6 h of incubation, the ability of the test compounds to bind to the PPARγ LBD and thus to displace the fluorescently labelled ligand was estimated from the decrease of the emission ratio 520 nm/495 nm upon excitation at 340 nm. Each data point represents the mean ± SEM from four independent experiments performed in duplicate.

As a partial PPARγ agonist, falcarindiol transactivated the luciferase reporter with an EC_50_ of 3.29 µM (95% CI: 2.33–4.64 µM) inducing a maximum of 3.26 (95% CI: 2.90–3.61)-fold activation, whereas the full PPARγ agonist pioglitazone displayed an EC_50_ of 0.41 µM (95% CI: 0.25–0.67 µM) and a maximum of 9.28 (95% CI: 8.08–10.48)-fold activation (Fig. 2A). The stimulating effect of 10 µM falcarindiol was blocked upon co-treatment with the PPARγ antagonist T0070907 confirming the PPARγ dependence of the observed activation (Fig. 2B). Noteworthy, the antagonistic effect of T007090907 was more pronounced when the receptor was activated with pioglitazone than with falcarindiol, an observation which is in agreement with previous studies demonstrating that the antagonistic potency of T007090907 is dependent on the type of ligand used to activate PPARγ [37]. Furthermore, co-treatment with falcarindiol blocked up to 70–80% of the stimulating effect of pioglitazone (1 µM) with an IC_50_ of 18.14 µM (95% CI: 14.32 to 22.97 µM), suggesting competition of the two compounds for the same binding site of the receptor (Fig. 2C).

### Receptor Binding and Molecular Modeling

To further demonstrate the direct receptor binding of falcarindiol we performed a TR-FRET competitive binding assay with purified human PPARγ-LBD (Fig. 2D). In this model falcarindiol and pioglitazone displayed *K_i_* values of 3.07 µM and 0.27 µM, respectively. Noteworthy, there was a very good correlation between the EC_50_ values obtained with falcarindiol and pioglitazone in the cell based luciferase reporter model (3.29 µM and 0.41 µM, respectively) and the *K_i_* values obtained in the receptor binding assay with purified PPARγ-LBD (3.07 µM and 0.27 µM, respectively).

**Figure 3:**
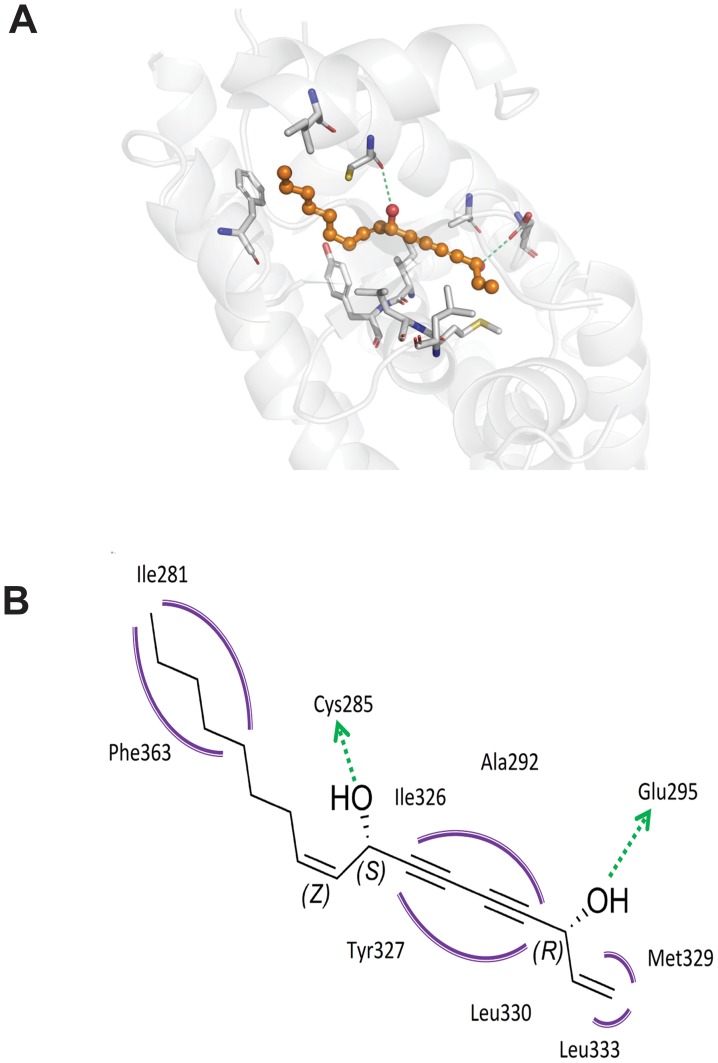
Proposed molecular binding mode of falcarindiol in the PPARγ LBD. Predicted binding mode of falcarindiol shown as 3D depiction in which predicted hydrogen-bonds are shown as dashed lines (**A**), and 2D depiction including chemical features of the interaction pattern derived from the docking pose (**B**). Chemical features in the 2D depiction are color-coded: green arrow – hydrogen-bond donor; magenta – hydrophobic contacts.

In order to gain insight into the molecular interaction of the tested polyacetylene-type PPARγ agonists with the receptor binding pocket of the protein, docking studies were performed (Fig. 3). The composition and overall structure of the PPARγ LBD have been analyzed previously, describing the PPARγ LBD with a Y-shaped topology [38]. The entrance includes several polar residues (e.g., Arg288, Glu291, Glu295, and Glu343), while the remaining two branches of the binding pocket, arm I and arm II, are composed mostly of hydrophobic residues, except a few moderately polar residues in arm I (e.g., Cys285, Ser289, His323, His449, and Tyr473) [38]. Consistent with the relatively similar structure (Fig. 1B) and bioactivity (Table 1) of the investigated polyacetylenes, all compounds (**1–6)** docked to the PPARγ LBD in a quite similar manner as depicted for falcarindiol (Fig. 3A and 3B). The molecular binding mode of the falcarindiol-type polyacetylene PPARγ partial agonists is proposed to consist of hydrophobic contacts of the ligand alkyl chains with residues of arm II (e.g., Ile281) and arm I (e.g., Ile326, Tyr327, Phe363) as well as residues of the entrance region (e.g., Ala292, Met329, Leu330, and Leu333). The hydroxyl groups form hydrogen bonds with the backbone amide of Cys285 in arm I, and the carboxylate group of Glu295 in the entrance (Fig. 3A and 3B).

### Effectiveness of Falcarindiol in Cell Models with Endogenous Expression of PPARγ

**Figure 4:**
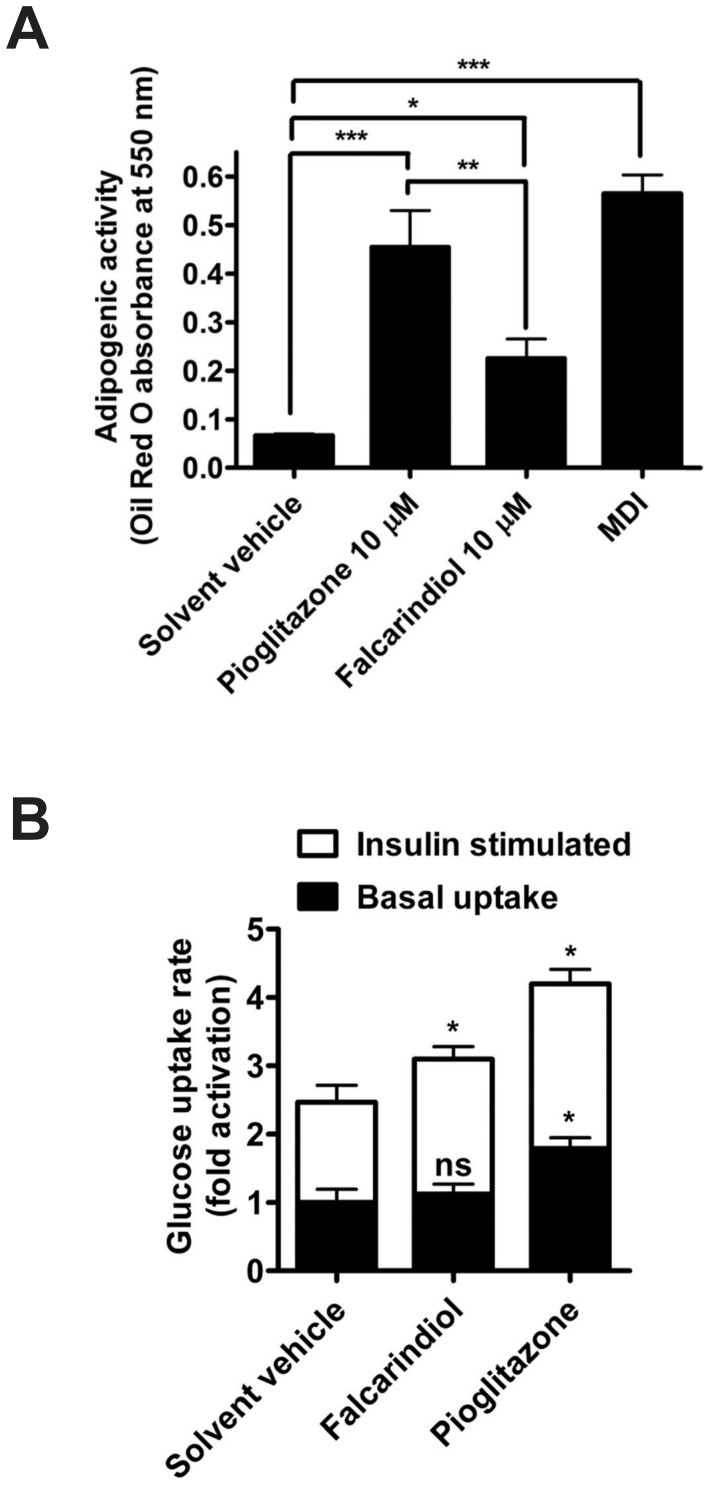
Assessment of adipogenicity and glucose uptake-enhancing properties of falcarindiol. (**A**) 3T3-L1 preadipocytes were differentiated to adipocytes as described in the *Materials and Methods* section for 10 days in the presence of pioglitazone, falcarindiol, solvent vehicle (0.1% DMSO), or a standard differentiation mixture (MDI; containing 10% FBS, 500 µM IBMX, 500 nM dexamethasone, and 1 µg/ml insulin) as a positive control. For an estimate of accumulated lipids, and thus for the adipogenic potential of the test compounds, Oil Red O staining and photometric quantification at 550 nm was performed. The data shown are means ± SD of three independent experiments. ***p<0.001, **p<0.01, *p<0.05 (ANOVA/Bonferroni). (**B**) 3T3-L1 mature adipocytes were incubated with falcarindiol (10 µM) or solvent vehicle (0.1% DMSO) for 48 h and 2-deoxy-D-(1H^3^)-glucose cellular uptake was determined for 10 min in the presence or absence (basal uptake) of insulin (500 pM). The data shown are means ± SEM of five independent experiments. *p<0.05 (compared to the solvent vehicle group; two-tailed paired *t*-test).

A main target site involved in the insulin-sensitizing action of the clinically used PPARγ agonists is the fat tissue [39], [40]. To further confirm the effectiveness of falcarindiol in a functionally relevant cell model with endogenous expression of PPARγ, we examined the effect of this compound on 3T3-L1 adipocyte differentiation and glucose uptake. As evident by the accumulation of lipid droplets and subsequent Oil Red O staining, the treatment with 10 µM falcarindiol induced the differentiation to adipocytes as expected, although to a smaller extent than pioglitazone at the same concentration (Fig. 4A). Furthermore, falcarindiol significantly enhanced the insulin-mediated glucose uptake in differentiated 3T3-L1 adipocytes (Fig. 4B).

## Discussion

In this study we identify polyacetylenes from *N. incisum* as representatives of a novel group of natural products activating PPARγ as partial agonists, and characterize in more detail the mode of action of the prototypical polyacetylene falcarindiol.

C(17)-polyacetylenes of the falcarindiol-type are found in many plants of the Apiaceae family, including commonly used vegetables and seasonings such as carrots, celery, fennel, dill, parsley, and parsnip [41], [42]. A variety of bioactivities have been reported so far for this compound class which may contribute to the health promoting effects of certain edible plants as well as medicinal plants used in traditional medicine [43]. The possibility that this compound class may modulate the activity of PPARγ, however, was so far not investigated. Since PPARγ is a key regulator of a number of pathways related to lipid and carbohydrate metabolism, this nuclear receptor represents an important pharmacological target for the treatment of diabetes type 2 and the metabolic syndrome. Furthermore, PPARγ appears to be a suitable target for modulation by diet or food supplements, since PPARγ activity was reported to be enhanced by a number of common food constituents or natural products present in certain seasonings or medicinal plants [44]–[46]. All polyacetylenes from *N. incisum* that were investigated in this study displayed a partial PPARγ agonism with a maximal activation several folds lower than the activation induced by the full PPARγ agonist pioglitazone (Table 1, Fig. 1B, and Fig. 2A). Docking studies (Fig. 3A and 3B) suggest receptor binding in a manner similar to other partial PPARγ agonists, which have been co-crystallized with the receptor, indicating that a hydrogen-bonding network formed by full agonists to residues of arm I is not formed in a comparable manner or even lacking in case of partial agonists [47], [48]. The partial agonism makes the polyacetylenes an interesting class of novel PPARγ ligands, since it is currently considered that the development of new partial agonists that do not activate PPARγ as strongly as the glitazone-type of full agonists might be a way to retain the needed pharmaceutical effectiveness with reduced side effects [49], [50]. Furthermore, the investigated polyacetylenes selectively activated PPARγ without activating PPARα or PPARβ/δ (Table 1). This is another rather favorable profile of action since the PPARγ agonists currently approved on the market are isoform-specific PPARγ activators, although there is some research evidence suggesting possible advantages for PPAR dual agonists or pan-agonists [50]. Very interestingly, previous studies have reported an antidiabetic activity of polyacetylenes isolated from *Bidens pilosa* upon oral administration in mice [51], possibly indicating effective modulation of PPARγ activity *in vivo*.

In summary, we report the identification of polyacetylenes as novel class of natural products activating PPARγ. The observed partial activation mode makes these compounds highly interesting PPARγ ligands, having the potential to be further explored as pharmaceutical leads or dietary supplements.

## References

[1] Lalloyer F, Staels B (2010) Fibrates, glitazones, and peroxisome proliferator-activated receptors. Arterioscler Thromb Vasc Biol 30: 894–899. 20393155 10.1161/ATVBAHA.108.179689 PMC2997800

[2] Hansen MK, Connolly TM (2008) Nuclear receptors as drug targets in obesity, dyslipidemia and atherosclerosis. Current opinion in investigational drugs 9: 247–255. 18311660

[3] Desvergne B, Michalik L, Wahli W (2006) Transcriptional regulation of metabolism. Physiological reviews 86: 465–514. 16601267 10.1152/physrev.00025.2005

[4] Evans RM, Barish GD, Wang YX (2004) PPARs and the complex journey to obesity. Nature medicine 10: 355–361. 10.1038/nm1025 15057233

[5] Schoonjans K, Martin G, Staels B, Auwerx J (1997) Peroxisome proliferator-activated receptors, orphans with ligands and functions. Current opinion in lipidology 8: 159–166. 9211064 10.1097/00041433-199706000-00006

[6] Sher T, Yi HF, McBride OW, Gonzalez FJ (1993) cDNA cloning, chromosomal mapping, and functional characterization of the human peroxisome proliferator activated receptor. Biochemistry 32: 5598–5604. 7684926 10.1021/bi00072a015

[7] Kliewer SA, Forman BM, Blumberg B, Ong ES, Borgmeyer U, et al (1994) Differential expression and activation of a family of murine peroxisome proliferator-activated receptors. Proceedings of the National Academy of Sciences of the United States of America 91: 7355–7359. 8041794 10.1073/pnas.91.15.7355 PMC44398

[8] Bardot O, Aldridge TC, Latruffe N, Green S (1993) PPAR-RXR heterodimer activates a peroxisome proliferator response element upstream of the bifunctional enzyme gene. Biochem Biophys Res Commun 192: 37–45. 8386511 10.1006/bbrc.1993.1378

[9] Ge K, Guermah M, Yuan CX, Ito M, Wallberg AE, et al (2002) Transcription coactivator TRAP220 is required for PPAR gamma 2-stimulated adipogenesis. Nature 417: 563–567. 12037571 10.1038/417563a

[10] Yao TP, Ku G, Zhou N, Scully R, Livingston DM (1996) The nuclear hormone receptor coactivator SRC-1 is a specific target of p300. Proceedings of the National Academy of Sciences of the United States of America 93: 10626–10631. 8855229 10.1073/pnas.93.20.10626 PMC38204

[11] Ricote M, Glass CK (2007) PPARs and molecular mechanisms of transrepression. Biochimica et biophysica acta 1771: 926–935. 17433773 10.1016/j.bbalip.2007.02.013 PMC1986735

[12] Desvergne B, Wahli W (1999) Peroxisome proliferator-activated receptors: nuclear control of metabolism. Endocr Rev 20: 649–688. 10529898 10.1210/edrv.20.5.0380

[13] Ziouzenkova O, Plutzky J (2004) Lipolytic PPAR activation: new insights into the intersection of triglycerides and inflammation? Current opinion in clinical nutrition and metabolic care 7: 369–375. 15192437 10.1097/01.mco.0000134358.46159.61

[14] Fredenrich A, Grimaldi PA (2005) PPAR delta: an uncompletely known nuclear receptor. Diabetes & metabolism 31: 23–27. 15803109 10.1016/s1262-3636(07)70162-3

[15] Abbott BD (2009) Review of the expression of peroxisome proliferator-activated receptors alpha (PPAR alpha), beta (PPAR beta), and gamma (PPAR gamma) in rodent and human development. Reproductive toxicology 27: 246–257. 18996469 10.1016/j.reprotox.2008.10.001

[16] Auwerx J (1999) PPARgamma, the ultimate thrifty gene. Diabetologia 42: 1033–1049. 10447513 10.1007/s001250051268

[17] Patsouris D, Muller M, Kersten S (2004) Peroxisome proliferator activated receptor ligands for the treatment of insulin resistance. Current opinion in investigational drugs 5: 1045–1050. 15535425

[18] Michalik L, Auwerx J, Berger JP, Chatterjee VK, Glass CK, et al (2006) International Union of Pharmacology. LXI. Peroxisome proliferator-activated receptors. Pharmacological reviews 58: 726–741. 17132851 10.1124/pr.58.4.5

[19] Semple RK, Chatterjee VK, O’Rahilly S (2006) PPAR gamma and human metabolic disease. The Journal of clinical investigation 116: 581–589. 16511590 10.1172/JCI28003 PMC1386124

[20] Lehmann JM, Moore LB, Smith-Oliver TA, Wilkison WO, Willson TM, et al (1995) An antidiabetic thiazolidinedione is a high affinity ligand for peroxisome proliferator-activated receptor gamma (PPAR gamma). The Journal of biological chemistry 270: 12953–12956. 7768881 10.1074/jbc.270.22.12953

[21] Subramanian V, Golledge J, Ijaz T, Bruemmer D, Daugherty A (2010) Pioglitazone-induced reductions in atherosclerosis occur via smooth muscle cell-specific interaction with PPAR{gamma}. Circulation research 107: 953–958. 20798360 10.1161/CIRCRESAHA.110.219089 PMC2963621

[22] Copland JA, Marlow LA, Kurakata S, Fujiwara K, Wong AK, et al (2006) Novel high-affinity PPARgamma agonist alone and in combination with paclitaxel inhibits human anaplastic thyroid carcinoma tumor growth via p21WAF1/CIP1. Oncogene 25: 2304–2317. 16331265 10.1038/sj.onc.1209267

[23] Peters JM, Shah YM, Gonzalez FJ (2012) The role of peroxisome proliferator-activated receptors in carcinogenesis and chemoprevention. Nature reviews Cancer 12: 181–195. 22318237 10.1038/nrc3214 PMC3322353

[24] Rubenstrunk A, Hanf R, Hum DW, Fruchart JC, Staels B (2007) Safety issues and prospects for future generations of PPAR modulators. Biochimica et biophysica acta 1771: 1065–1081. 17428730 10.1016/j.bbalip.2007.02.003

[25] Bhalla K, Hwang BJ, Choi JH, Dewi R, Ou L, et al (2011) N-Acetyl Farnesyl cysteine is a novel class of PPAR{gamma} ligand with partial and full agonist activity in vitro and in vivo. J Biol Chem 286: 41626–41635. 21979952 10.1074/jbc.M111.257915 PMC3308872

[26] Agrawal R, Jain P, Dikshit SN (2012) Balaglitazone: A Second Generation Peroxisome Proliferator-Activated Receptor (PPAR) Gamma (gamma) Agonist. Mini reviews in medicinal chemistry 12: 87–97. 22372600 10.2174/138955712798995048

[27] Chandalia A, Clarke HJ, Clemens LE, Pandey B, Vicena V, et al (2009) MBX-102/JNJ39659100, a Novel Non-TZD Selective Partial PPAR-gamma Agonist Lowers Triglyceride Independently of PPAR-alpha Activation. PPAR research 2009: 706852. 19404482 10.1155/2009/706852 PMC2673481

[28] Newman DJ, Cragg GM (2012) Natural Products As Sources of New Drugs over the 30 Years from 1981 to 2010. Journal of natural products 75(3): 311–335. 22316239 10.1021/np200906s PMC3721181

[29] Liu X, Jiang S, Xu K, Sun H, Zhou Y, et al (2009) Quantitative analysis of chemical constituents in different commercial parts of Notopterygium incisum by HPLC-DAD-MS. Journal of ethnopharmacology 126: 474–479. 19781618 10.1016/j.jep.2009.09.011

[30] Zschocke S, Lehner M, Bauer R (1997) 5-Lipoxygenase and cyclooxygenase inhibitory active constituents from Qianghuo (Notopterygium incisum). Planta medica 63: 203–206. 9265192 10.1055/s-2006-957653

[31] Graham FL, van der Eb AJ (1973) A new technique for the assay of infectivity of human adenovirus 5 DNA. Virology 52: 456–467. 4705382 10.1016/0042-6822(73)90341-3

[32] Chung JY, Hah JM, Cho AE (2009) Correlation between performance of QM/MM docking and simple classification of binding sites. Journal of Chemical Information and Modeling 49: 2382–2387. 19799409 10.1021/ci900231p

[33] Zhang H, Xu X, Chen L, Chen J, Hu L, et al (2011) Molecular determinants of magnolol targeting both RXRalpha and PPARgamma. PLoS One 6: e28253. 22140563 10.1371/journal.pone.0028253 PMC3226690

[34] Wolber G, Langer T (2005) LigandScout: 3-D pharmacophores derived from protein-bound ligands and their use as virtual screening filters. J Chem Inf Model 45: 160–169. 15667141 10.1021/ci049885e

[35] Bradford MM (1976) A rapid and sensitive method for the quantitation of microgram quantities of protein utilizing the principle of protein-dye binding. Analytical biochemistry 72: 248–254. 942051 10.1016/0003-2697(76)90527-3

[36] Cheng Y, Prusoff WH (1973) Relationship between the inhibition constant (K1) and the concentration of inhibitor which causes 50 per cent inhibition (I50) of an enzymatic reaction. Biochem Pharmacol 22: 3099–3108. 4202581 10.1016/0006-2952(73)90196-2

[37] Waku T, Shiraki T, Oyama T, Maebara K, Nakamori R, et al (2010) The nuclear receptor PPARgamma individually responds to serotonin- and fatty acid-metabolites. The EMBO journal 29: 3395–3407. 20717101 10.1038/emboj.2010.197 PMC2957204

[38] Zoete V, Grosdidier A, Michielin O (2007) Peroxisome proliferator-activated receptor structures: ligand specificity, molecular switch and interactions with regulators. Biochimica et biophysica acta 1771: 915–925. 17317294 10.1016/j.bbalip.2007.01.007

[39] Tontonoz P, Spiegelman BM (2008) Fat and beyond: the diverse biology of PPARgamma. Annual review of biochemistry 77: 289–312. 10.1146/annurev.biochem.77.061307.091829 18518822

[40] Anghel SI, Wahli W (2007) Fat poetry: a kingdom for PPAR gamma. Cell Res 17: 486–511. 17563755 10.1038/cr.2007.48

[41] Soltoft M, Eriksen MR, Trager AW, Nielsen J, Laursen KH, et al (2010) Comparison of polyacetylene content in organically and conventionally grown carrots using a fast ultrasonic liquid extraction method. Journal of agricultural and food chemistry 58: 7673–7679. 20560673 10.1021/jf101921v

[42] Christensen LP, Brandt K (2006) Bioactive polyacetylenes in food plants of the Apiaceae family: occurrence, bioactivity and analysis. Journal of pharmaceutical and biomedical analysis 41: 683–693. 16520011 10.1016/j.jpba.2006.01.057

[43] Christensen LP (2011) Aliphatic C(17)-polyacetylenes of the falcarinol type as potential health promoting compounds in food plants of the Apiaceae family. Recent patents on food, nutrition & agriculture 3: 64–77. 10.2174/2212798411103010064 21114468

[44] Fakhrudin N, Ladurner A, Atanasov AG, Heiss EH, Baumgartner L, et al (2010) Computer-aided discovery, validation, and mechanistic characterization of novel neolignan activators of peroxisome proliferator-activated receptor gamma. Mol Pharmacol 77: 559–566. 20064974 10.1124/mol.109.062141 PMC3523390

[45] Christensen KB, Petersen RK, Kristiansen K, Christensen LP (2010) Identification of bioactive compounds from flowers of black elder (Sambucus nigra L.) that activate the human peroxisome proliferator-activated receptor (PPAR) gamma. Phytotherapy research : PTR 24 Suppl 2S129–132. 20222152 10.1002/ptr.3005

[46] Marion-Letellier R, Dechelotte P, Iacucci M, Ghosh S (2009) Dietary modulation of peroxisome proliferator-activated receptor gamma. Gut 58: 586–593. 19017686 10.1136/gut.2008.162859

[47] Pochetti G, Godio C, Mitro N, Caruso D, Galmozzi A, et al (2007) Insights into the mechanism of partial agonism: crystal structures of the peroxisome proliferator-activated receptor gamma ligand-binding domain in the complex with two enantiomeric ligands. The Journal of biological chemistry 282: 17314–17324. 17403688 10.1074/jbc.M702316200

[48] Guasch L, Sala E, Valls C, Blay M, Mulero M, et al (2011) Structural insights for the design of new PPARgamma partial agonists with high binding affinity and low transactivation activity. Journal of computer-aided molecular design 25: 717–728. 21691811 10.1007/s10822-011-9446-9

[49] Yumuk VD (2006) Targeting components of the stress system as potential therapies for the metabolic syndrome: the peroxisome-proliferator-activated receptors. Ann N Y Acad Sci 1083: 306–318. 17148746 10.1196/annals.1367.019

[50] Chang F, Jaber LA, Berlie HD, O’Connell MB (2007) Evolution of peroxisome proliferator-activated receptor agonists. Ann Pharmacother 41: 973–983. 17519293 10.1345/aph.1K013

[51] Chien SC, Young PH, Hsu YJ, Chen CH, Tien YJ, et al (2009) Anti-diabetic properties of three common Bidens pilosa variants in Taiwan. Phytochemistry 70: 1246–1254. 19683775 10.1016/j.phytochem.2009.07.011

